# Spatio-Temporal Human Grip Force Analysis via Sensor Arrays

**DOI:** 10.3390/s90806330

**Published:** 2009-08-12

**Authors:** Dieter F. Kutz, Alexander Wölfel, Tobias Meindl, Dagmar Timmann, Florian P. Kolb

**Affiliations:** 1 Department of Physiological Genomics, Institute of Physiology, University of Munich, Pettenkoferstr. 12, 80336 Munich, Germany; E-Mails: woelfela@googlemail.com (A.W.); meindl.tobias@googlemail.com (T.M.); f.kolb@lmu.de (F.P.K.); 2 Department of Neurology, University of Duisburg-Essen, Hufelandstr. 55, 45138 Essen, Germany; E-Mail: dagmar.timmann-braun@uni-duisburg-essen.de (D.T.)

**Keywords:** prehension, multi-digit grip, dexterity, disability evaluation, hand and finger strength, rehabilitation

## Abstract

This study describes a technique for measuring human grip forces exerted on a cylindrical object via a sensor array. Standardised resistor-based pressure sensor arrays for industrial and medical applications have been available for some time. We used a special 20 mm diameter grip rod that subjects could either move actively with their fingers in the horizontal direction or exert reactive forces against opposing forces generated in the rod by a linear motor. The sensor array film was attached to the rod by adhesive tape and covered approximately 45 cm^2^ of the rod surface. The sensor density was 4/cm^2^ with each sensor having a force resolution of 0.1 N. A scan across all sensors resulted in a corresponding frame containing force values at a frame repetition rate of 150/s. The force value of a given sensor was interpreted as a pixel value resulting in a false-colour image. Based on remote sensed image analysis an algorithm was developed to distinguish significant force-representing pixels from those affected by noise. This allowed tracking of the position of identified fingers in subsequent frames such that spatio-temporal grip force profiles for individual fingers could be derived. Moreover, the algorithm allowed simultaneous measurement of forces exerted without any constraints on the number of fingers or on the position of the fingers. The system is thus well suited for basic and clinical research in human physiology as well as for studies in psychophysics.

## 1. Introduction

The ability to grasp objects enables humans to perform a wide range of manipulative movements. Skilled control of prehensile finger forces is an essential feature of tool use in daily life. In healthy subjects, grip force is scaled accurately to object properties so as to prevent a hand-held object from slipping [1–3]. Impaired hand function is a frequent observation in movement disorders. The characteristics of impaired finger force control include inefficient grip force scaling and imprecision of the temporal coupling between grip and load force profiles. Grip force analysis is a highly sensitive method for detecting even subtle impairments of finger force control or to document impaired dexterity and its rehabilitation in movement disorders [4,5].

To date, several techniques have been developed for measuring multi-grip forces simultaneously [6–14]. The most common measurement system employs three or more force/torque transducers in a fixed arrangement [6,7,9,10,14–16]. Although these systems measure force very accurately, their disadvantage for studies on patients with motor disorders is the fixed, and thus inconvenient, arrangement of finger positions as well as the reduced contact area. A different method of measuring grip forces has been presented by Pylatiuk and colleagues [13]. They attached sensors directly to the finger and palmar skin with double-sided adhesive tape, leading to altered skin sensitivity. Acute skin sensitivity, however, is required for the internal representation of the physical object properties [17–20]. Pylatiuk’s system is suitable for studies on prehension of force grips but less appropriate for studies on grip forces of precision grips. An early publication using sensor arrays for grip force measurements [8] described a system specialised for quantifying grip activity during handwriting. In this system a force-measuring film was attached to a writing utensil. Due to its specialization this system had low spatial resolution and did not allow the separation of different finger grip forces over time.

The present study presents a technique employing a sensor array to measure grip forces exerted by the human hand on an object. The use of the sensor array enabled grip force measurements over a large contact area (up to 170 cm^2^) with high spatial resolution (5.08 mm sensor distance), good force resolution (0.1 N) and reasonable temporal resolution (150 Hz). In addition, the forces exerted by any number of fingers could be measured simultaneously without any constraints on finger position. This is essential for studying patients with impaired dexterity. Our main research interest was the evaluation of grasping abilities of patients with cerebellar disease. For this reason, we created a new grasping task in which subjects had to pull an initially blocked and then unexpectedly released grip rod, which is a task comparable with picking a raspberry. Picking a raspberry requires perfectly adjusted, increasing grip forces to pull the raspberry off the bush without squashing it. This, however, was very difficult for cerebellar patients. The representative data presented here demonstrate the ability of the measurement system, and are not intended as a statement on grasping dexterities of patients with cerebellar disease.

## 2. Materials and Methods

### 2.1. Grip Rod and Force-Measuring Film

Grip rod, force-measuring film and algorithms have been described in detail elsewhere [11,12]. A brief but comprehensive description of the system follows. The grip rod comprised a cylindrical metal bar (20 mm diameter), a linear motor (type: STA2505, Copley Controls, Canton, MA, USA) that could move the grip rod up to 100 mm axially and horizontally, a linear potentiometer to measure the position (type: REM 13-200-K, Megatron Elektronik, Putzbrunn, Germany) and a force transducer to measure the force exerted along the rod (type: U9B, Hottinger Baldwin Messtechnik, Darmstadt, Germany). In the pull or push direction the maximal force was hardware-limited to 25 N. The motor was controlled by custom-written software using LabVIEW (v. 8.2, National Instruments, Austin, TX, USA). The static friction of the grip rod was 0.6 N and the dynamic friction 0.3 N. To produce a 6-N square pulse the motor reacted in less than 15 ms.

**Figure 1.**
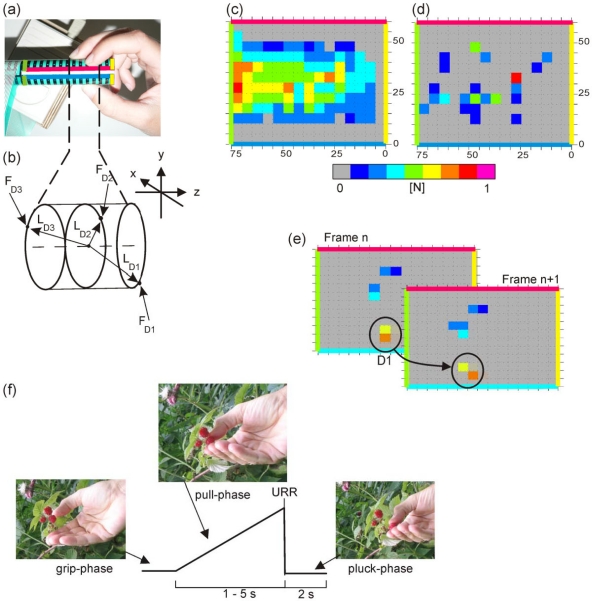
(a) Top view of the grip rod with the attached force-measuring film. (b) Determination of a rod slice element D1 = thumb, D2 = index finger, D3 = middle finger, F_D1–3_ = orthogonal grip forces of D1–3, L_D1–3_ = lever of D1–3. (c) Uncompensated noise due to bending without finger contact. (d) Remaining mean noise after Tekscan-software reduction. (e) Pairwise correlation of significant finger forces. For (c–e): Each square represents sensor value of the sensor array and is seen as a pixel of a sensed image. (f) Schema of the “pick the raspberry task” with determing the different corresponding phases.

A standardised resistor-based pressure sensor array (type: 3000/HOT, Tekscan, MA, USA) was used as force-measuring film. It contained sensors in a rectangular order with a distance of 5.08 mm in each direction. The force range of each sensor was 0.89–13.3 N. Some 45 cm^2^ of the rod surface was covered by the force-measuring film which was attached on one side to the rod with double-sided adhesive tape. The measuring film was bent from above around the rod so that the edges in axial direction were situated at the top (see Figure 1a). The used areas of the measuring film contained 180 sensors, 15 columns along the rod axis covering 75 mm of the grip rod length and 12 rows orthogonal to them covering 61 mm of the circumference. This allowed force measurements to be performed with extended fingers. Bending the measurement film around the rod elicited inhomogeneous noise with forces up to 1 N at a given single sensor (see Figure 1c). In the data image of Figures 1c–d the two edges of the sensor array in axial orientation (corresponding to Figure 1a) are indicated as red and blue lines, respectively. The grip rod end is indicated as a yellow line at the right edge of the image and the edge of used sensor area is indicated by the green line at the left border of the image. The force values were measured synchronously and stored as an image frame using F-Scan software (v. 5.24, Tekscan) on a desktop computer. Data were sampled at 150 frames/s.

### 2.2. Detection of Grip Forces and Correlation of Finger Positions with Forces Significantly Detected

Values recorded via the sensor array at any time t_i_ are seen as pixels of a sensed image at t_i_ and are presented as a false-colour image (e.g., Frame n and n+1 in Figure 1e). Based on this idea, significant grip forces [11] was achieved by a modification of Rogerson’s algorithm for change detection in remotely sensed images [21].

The recording software is able to subtract a noise image from any recorded image. Nevertheless, measuring force values without any fingers in contact with the grip rod and applying the subtraction algorithm to the recorded values revealed a noticeable level of remnant noise in the data. This remaining noise level had to be taken into account when the grip rod was touched softly and consequently, the resulting signal-to-noise ratio was low. This consideration is taken into account in the formulation of Equation 1 and the remaining mean noise level was measured separately in a noise film of at least 300 frames (Figure 1d). The algorithm was divided in three steps: (1) transformation of the image F_i_ from a N(μ,σ)-distribution in a N(0,1)-distribution, (2) smoothing the data with a Gaussian kernel to detect sub regions of local spatial association, (3) detection of significant pixel with Bonferroni correction [21,22].

1. N(μ,σ) → N(0,1)
  (1) $$\begin{array}{c} R_{i}\left(p_{j}\right)=F_{i}\left(p_{j}\right)-\bar{\mathrm{Noise}\left(p_{j}\right)}\\ {\mathrm{nR}}_{i}\left(p_{j}\right)=\{\begin{array}{cc} \frac{R_{i}\left(p_{j}\right)}{S.D.\left(R_{i}\right)} & \mathrm{if}\;S.D.\;\left(R_{i}\right)>95\%\;\;c.v.\\ \frac{R_{i}\left(p_{j}\right)}{95\%\;\;c.v.} & \mathrm{otherwise} \end{array} \end{array}$$
  *R_i_* (*p_j_*) = residual of pixel *p_j_* in frame *F_i_*; *F_i_* (*p_j_*) is the raw value of pixel *p_j_*; $\bar{\mathrm{Noise}\left(p_{j}\right)}$ = mean noise at pixel *p_j_*, measured separately across at least 300 frames of the noise film (see Figure 1d). *n R_i_* (*p_j_*) = normalised residual of pixel *p_j_*, in frame *F_i_*. *S. D.* (*R_i_*) = standard deviation of the residual of frame *F_i_*. 95% c.v. = 95% upper confidence value of the system noise level measured in the noise film.
2. Smoothing each frame:
  Each frame is smoothed with a Gaussian kernel *K(p_j_,p_k_)* to improve the signal to noise ratio. The values *z_j_* of the smoothed normalised residual frame represent local statistics that have the purpose of detecting sub regions of local spatial association. The parameter *σ* (in pixel) is chosen to match the scale at which spatial association exists (Equations 3 and 4 from [21]).
  (2) $$z_{j}=\frac{\sum {\mathrm{nR}}_{i}\left(p_{j}\right)*K\left(p_{j},\;p_{k}\right)}{\sqrt{\sum \left(K{\left(p_{j},\;p_{k}\right)}^{2}\right)}}$$
  with *nR_i_*(*p_j_*) = normalised residual of pixel *p_j_* in Frame *F_i_* and *K*(*p_j_,p_k_*) as:
  K(pj, pk)=1πe(−d(pj, pk)22σ2)
  where *d*(*p_j_,p_k_*) = distance between pixel p_j_ and p_k_.
3. *Significant pixel detection*. The third step was to detect the significance of force as a pixel in a frame, whereby the number of tests depended on the total number of pixels and the value of σ^2^ of the Gaussian kernel (Equation 2). The larger the value of σ^2^, the greater was the spatial correlation between local tests. Rogerson provided an approximation (Equation 3) that allowed direct calculation of the observed maximum local statistics [21,22]:
  (3) $$M^{*}\approx \sqrt{-\sqrt{\pi }\ln\left(\frac{4\alpha \left(1+0.81\sigma^{2}\right)}{A}\right)}$$
  by using α = 0.05, σ = 0.5, A = 12 × 15 pixel = 180 resulted in M* = 3.425. Any value *z_j_* in the smoothed normalised residual frame exceeding M* is significant at the 5%-level of first error.

### 2.3. Pairwise Correlation of Single-Finger Forces between Frames

Position and force-values were assigned semi-automatically to individual fingers [11]. The algorithm assumes that the fingers were not crossed, that changes in finger position between frames were small, and that changes in finger position were continuous. In at least one frame significant data were assigned manually to the fingers. Starting from this position the algorithm assigned data to the fingers automatically up to the start/end of the complete sequence or the next starting position given by the user. The algorithm correlated pairwise the assigned position between neighbouring frames. This process was divided into three steps (Figure 1e):

1. By using the “Flood-fill-algorithm”, recognised coherent areas were marked (see http://en.wikipedia.org/wiki/Flood_fill). In this way significant pixels situated horizontally or vertically adjacent were combined (e.g., circle in Frame n of Figure 1e). The weighted centre of force was calculated for each marked area.
2. Centres of areas lying inside a given distance (intraframe distance: 1.645 pixel) were considered to be of the same origin and were combined. In this way combined areas contacted at their corners only (e.g., circle in Frame n+1 of Figure 1e).
3. Subsequently, the final weighted centres were compared and combined with the centres of the previous frame. The maximum distance between centres of the same finger was here 0.8 pixel (interframe distance). An example is given in Figure 1e. From the position of finger D1 (thumb) in Frame n followed the position of D1 in Frame n + 1.

The force detection algorithm as well as the position correlation algorithm were written in Yorick interpreter language (v. 1.6.0.2, [23]).

### 2.4. Dynamic Torque Analysis

The term “torque” is used interchangeably in mechanics. In this study “torque” was used to designate a force moment resulting from normal finger forces which would tend to deviate the rod from the pull-direction and represent losses that subjects try unconsciously to minimise.

From the finger positions (see Figure 1a), a gripped rod slice element and its centre of mass (CoM) were defined. The rod slice element was the part of the rod between the remotest fingers (e.g., rod part between fingers D1 and D3 in Figure 1b). Levers were derived from the position of individual fingers and the centre of mass of the rod slice element. Individual torques were given by the vector product of finger force and the lever, defined by the distance from the position of the finger on the rod on which the force was exerted to the centre of mass of the rod slice element. Torques of the individual fingers were calculated at each time (Equation 4a). From these data a total torque function was calculated over time for each finger (Equation 4b). The torques described here were situated all orthogonally to the pull axis in the x/y-plane according to Figure 1b. Hence, the rod would deviate from the pull-axis. However, given that the rod is blocked the torque produces a virtual deviation only.

(4a) $$M_{D_{i}}\;(t)=L_{D_{i}}\;(t)\times F_{D_{i}}\;(t)$$

(4b) $$M_{\sum }\;(t)=\sum_{i}M_{D_{i}}\;(t)$$

with *M_Di_*(*t*): torque, *L_Di_*(*t*): lever, *F_Di_*(*t*): grip force of individual finger *D_i_*, *M_Σ_*(*t*): sum of all torques *M_Di_*(*t*) at time *t*.

### 2.5. “Pick the Raspberry” Task

The “pick the raspberry task” is a precision grip task that requires continuous adjustments of grip and pull forces [12]. Subjects were seated comfortably on a height-adjustable chair facing the rod. The upper arm was held almost vertical and the forearm was flexed at the elbow joint 90° in the sagittal plane. The forearm was in a neutral position between pronation and supination. Subjects were asked to pull the rod horizontally with a three-finger precision grip (see Figure 1a). Each trial was self-paced by the subjects and its start was detected when a pull force velocity threshold of 0.5 N/s was exceeded. After a randomly variable interval (1–5 s) the rod was released (unpredictable rod release/URR). After rod release, the rod moved due to the subject’s pull force. Subjects were asked to stop the pull movement of the rod with low grip force. The interval from recording start until the detected trial start was termed the grip phase, the following interval until the URR was termed the pull phase and the last interval was termed the pluck phase (Figure 1f). If during pull-phase the velocity (i.e., the pull force rate) exceeded an upper limit (5 N/s) subjects were informed by a red LED in front of them. The inter-trial-interval was in the range of 10 s to 15 s. Prior to recording, subjects were allowed at least 10 trials for practice. The time course of the corresponding and natural task and the requested increase of the pull force are shown in Figure 1f.

### 2.6. Simulation of the “Pick the Raspberry” Task

The reliability of the force-measuring film was evaluated by simulating the mean behaviour of a control subject during the “pick the raspberry” task. For this purpose a continuously increasing motor-driven force (2.5–10 N) was applied to the sensor array over an area of approximately 300 mm^2^ for 4.5. After the maximal force value of 10 N the motor-driven force was released immediately. Force range and duration of the simulation represents the average development of force of subjects participating in the experiments. From the results of the simulation was the detection threshold of the sensors and a correction function calculated. The correction function was used for the calculation of the data shown in Figures 3–5.

### 2.7. Subjects and Patients

This study was performed with the permission of the ethics committee of the Ludwig in Maximilians-University of Munich (354-06). A total of eleven patients with cerebellar disease and sixteen healthy subjects participating after giving written informed consent were used to test thoroughly the measurement system. For demonstration purposes of the system under normal conditions (CTRL) and in typical situations of patients with motor disorder of the upper limbs (CBL1-3) characteristic results from one healthy subject (CTRL: female, 28 years, right handed) and from three cerebellar patients (CBL1: female, 41 years, right handed, sporadic adult onset ataxia; CBL2: male, 45 years, right handed, autosomal dominant cerebellar ataxia, ADCA (III); CBL3: female, 70 years, right handed, sporadic adult onset ataxia) are shown.

## 3. Results and Discussion

This study presents a technique employing a sensor array for measuring grip forces exerted by the human hand on a cylindrical object. Force values of all sensors were interpreted as pixel values of a false-colour image. The detection of grip forces was based on a modified change detection algorithm for remote sensed image analysis which distinguished significant force-representing pixels from those affected by noise [11]. The position of identified fingers was tracked in subsequent frames to construct spatio-temporal grip force profiles of an individual finger. The algorithm allowed simultaneous measurement of forces exerted without any constraints on the number of fingers or on the position of the fingers. The system is thus well suited for studying grip forces exerted by both healthy subjects and patients with impaired dexterity.

### 3.1. Reliability test of the Force-Measuring Film

**Figure 2.**
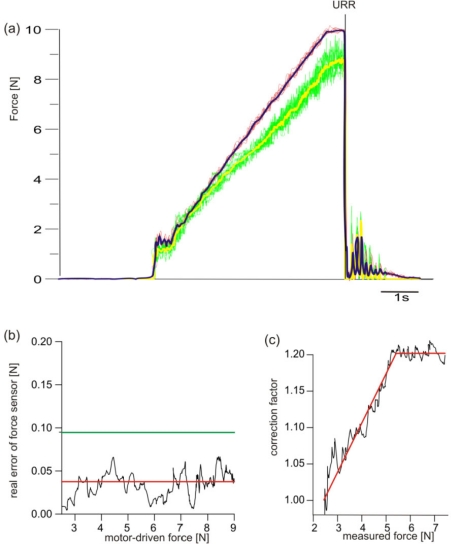
Simulation of force change during the “pick the raspberry” task. (a) Motor-driven forces of ten repetitions (red lines) and mean value (blue line) are shown as well as the measured forces (green lines) and their mean value (yellow line). (b) Real error of force sensor over motor-driven force with mean value (red line) and upper 1%-confidence band (green line). (c) Compensatory correction function for measured forces.

The “pick the raspberry” task was simulated to evaluate the reliability of the force sensors (see Section 2.6). The results from ten repetitions are shown in Figure 2a. The red lines show the motor-driven forces; the green lines show the measured forces. The sensor array follows the motor-driven forces sufficiently precisely over the range of forces of interest, as can be seen by the averaged motor-driven and measured forces (blue and yellow lines, respectively). After reaching the maximal force value of 10 N the motor-driven force was released immediately. This was followed by a vibration of the system due to technical constraints. Even this reaction was measured adequately by the sensor array.

While the motor-driven forces were exposed to noise, the real error of the sensor array was determined by following equation:

real error=SEM(motor−driven force)−SEM(measured force)

with SEM(measured force), SEM(motor-driven force): standard error of the mean of the measured or motor-driven force, respectively.

Figure 2b shows the result. The real error of the sensor array was independent of the motor-driven force in the range of interest. The mean error was 35.4 mN (red line in Figure 2b). Setting a 1%-level for detecting significant changes of force values an upper confidence value of 91.3 mN (green line in Figure 2b) was calculated. Hence, the detection threshold of the sensor array in this application can be given as 0.1 N. Since the sensors underestimate the effective motor-driven forces a correction function was introduced. The correction function, which increased linearly within the force range 2.5–5 N, remained stable at 1.2 for values above 5 N (Figure 2c).

### 3.2. Spatio-Temporal Profiles of Individual Finger Grip Force Development

**Figure 3.**
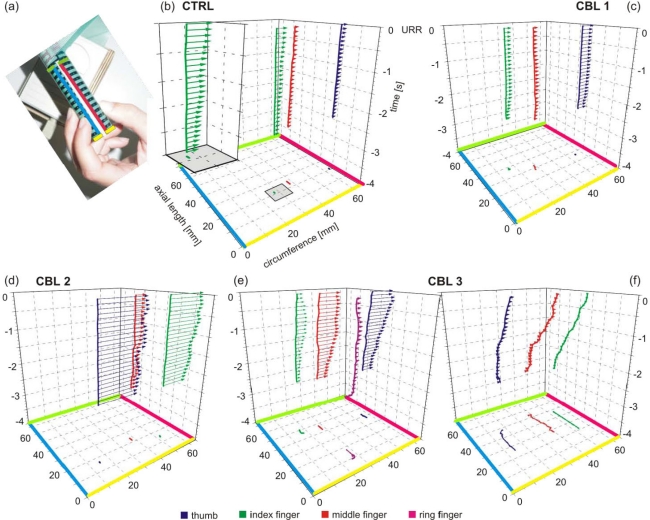
Spatio-temporal grip profiles of a healthy subject and three patients with degenerative cerebellar disease. Each plot shows the data of a single trial from 3.5 s before rod release up to URR at t = 0. (a) Demonstration of the requested three-finger grip. (b–f) Space-time cube of grip forces with 3D-graph of finger position and grip force vectors at discrete time intervals (see text). (b) Grip forces of a healthy subject (CTRL), with the inset showing spatio-temporal grip profile of the index finger only. (c) Grip forces of a cerebellar patient (CBL1) with moderate hypermetric grip forces. (d) Grip forces of a cerebellar patient (CBL2) with strong hypermetric grip forces. (e) Grip forces of a cerebellar patient (CBL3) with hypermetric grip forces and the use of a four-finger instead of a three-finger grip (left hand). (f) Grip force data of the same patient as in (e) with hypometric grip forces sliding with the fingers along the rod (right hand). For b–f: blue - thumb, green - index finger, red–middle finger, magenta–ring finger.

The sensor array described here allows the measurement of human grip forces of any number of fingers exerted at any position of the grip rod. This arrangement allowed the development of individual grip forces and the change of positions of all gripping fingers to be studied. A three-finger grip is demonstrated as well as the area of sensor array used, limited by coloured edges (Figure 3a) as described in Section 2.1.

Based on the algorithm (see Sections 2.3–2.4) spatio-temporal profiles of individual finger forces were calculated. Each individual finger was described by a series of data four-tuples containing the position of finger on the rod, the time of recording, and the measured force. This 4D-data problem is visualised as a space-time cube of grip forces with x-axis as the circumference, y-axis as axial length of the rod, and z-axis as time (Figures 3b–f). The sensory-array is unrolled and the edges of the used array are shown in the same colours as in Figure 3a. The position of each finger of a control subject (CTRL) over time is illustrated as a 3D-curve in space and time (Figure 3b). In addition, the individual finger positions are projected also in the x/y-plane. Grip forces of individual fingers are shown as arrows which length represents the measured grip force. For visualisation purposes grip forces are shown at time intervals of 100 ms. The arrows always start at the space-time point of measurement and are always directed along the y-axis. The projection in the x/y-plane is omitted for clarity.

Spatio-temporal profiles were constructed from single trials of different subjects. They are shown for an analysis time from 3.5 s preceding the unpredictable rod release (URR) to the URR (t = 0) in Figure 3b–f. The control subject produced a stable, isometric three-finger grip as can be derived from the projection of the position in the x/y-plane (Figure 3b). The grip was stabilised mainly by the thumb (blue line) and the index finger (green line) with additional assistance from the middle finger (red line). The continuous, moderate increase of grip forces over time is illustrated as in the additional space-time cube with the grip force vectors of the index finger only (Figure 3b).

Patients with cerebellar degeneration showed a different picture. CBL1 showed a pattern similar to that of the healthy subject but had clearly higher grip forces and a larger finger aperture (see the distance of the fingers in the x/y-plane, Figure 3c). CBL2 exerted high grip forces over the total analysis time (Figure 3d). This latter patient stabilised the grip with the thumb and the index finger, as did CTRL, although he exerted a high grip force with the middle finger as well. Another patient (CBL3, Figure 3e,f) showed completely different behaviour depending on the hand used. With the left hand, the patient produced hypermetric grip forces and used the ring finger as well (magenta line in Figure 3e). In contrast, when using the right hand, the patient produced hypometric grip forces, just touching the rod and sliding along the rod with his fingers.

Consequently, measurement systems with fixed finger positions are suitable for studies with healthy subjects, but are of limited use for patients with motor disorders. For the latter group, measurement systems with enlarged contact area and reduced finger aperture are essential [4,5,11,12]. Although all subjects exhibit different strategies when gripping the rod and following the task instruction, all corresponding grip forces have to be recorded by the measuring system. The data presented in Figure 3 yield the potential of the sensor array technique, with the effectiveness of our system shown at the best in Figure 3e,f. When using the left hand CBL3 employed four fingers instead of three and when using the right hand the patient did not exert sufficient grip forces to prevent sliding along the rod. This kind of behaviour can be studied only via a sensor array-based system as introduced in this study. Grip force measuring systems with fixed finger positions are used frequently [6,7,9,10,14–16] but are unable to detect losing a test object, as occurred with CBL3 (Figure 3e), or the employment of an additional finger (Figure 3e).

### 3.3 Dynamic Development of Torque

**Figure 4.**
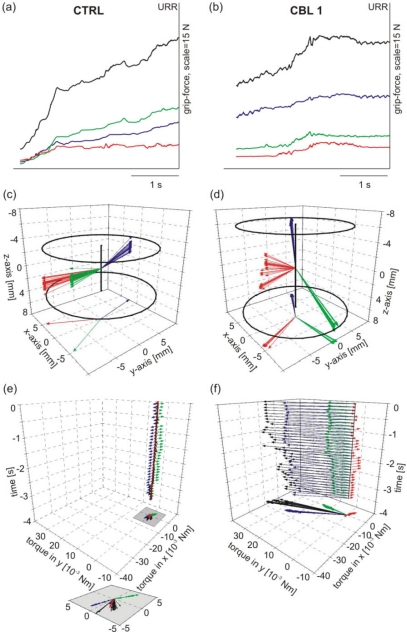
Dynamic torque development of a healthy subject (CTRL, left column) and a cerebellar patient (CBL 1, right column). (a, b) Grip force development of individual finger grip forces and total grip force (black line); same data as in Figure 3 b,c. (c, d) Levers of individual fingers. (e, f) Development of individual finger torques and total torque (black vectors). The inset in Figure 4e shows the extension of the projected torque vectors shown in the grey area. For a–f: colour convention as in Figure 3.

The individuality with which different subjects solve the task is illustrated in Figure 3. Although different pull forces were recorded they result from the same total grip forces. The development of grip forces during the trials illustrated in Figure 3b,c obtained from CTRL and CBL1 are compared now (Figure 4a,b). The total grip forces (black line) of both subjects reached approximately the same maximal value at URR. However, at URR CTRL produced 20 % more pull force than CBL1 (data not shown). This indicates that the grip of CTRL was more efficient than that of CBL1. Analysing the development of individual grip forces it can be seen that the CTRL had an equilibrated force relation over all fingers (thumb–blue line, index finger–green line, middle finger–red line) whereas the patient tended to employ the thumb as the main force-exerting finger (approx. 50% of total grip force at URR) and used the other fingers less effectively. Individual grip forces alone were not sufficient to explain the difference in economy of the two different grips. Due to the finger position the individual finger-grip forces produced not only pull force in horizontal direction but also torques, which would cause the grip rod to deviate from the pull axis. These torques are losses and are uneconomic in the biomechanical sense.

From the finger positions on the rod, we calculated the centre of mass of the rod slice element touched and the corresponding levers of each finger. Figure 4c,d shows the individual levers of the data presented in Figure 3b,c, respectively. The maximal rod slice element given by the finger positions is determined by 2 circles within the work space cube (Figure 4c,d) with a clearly larger rod slice element for the patient (Figure 4d) than for the control subject (Figure 4c). From lever and grip forces the torque of each individual finger can be calculated as well as the total torque (see Equation 4b, Section 2.4). The individual torques (coloured vectors) and the total torque vector (black vector) for both subjects are illustrated in Figures 4e,f. In addition, the projections of the torques in the x/y-plane are shown. The individual torques as well as the total torque of the control subject (Figure 4e) are clearly smaller than those of the cerebellar patient (Figure 4f) indicating a more economic grip than that of the patient.

**Figure 5.**
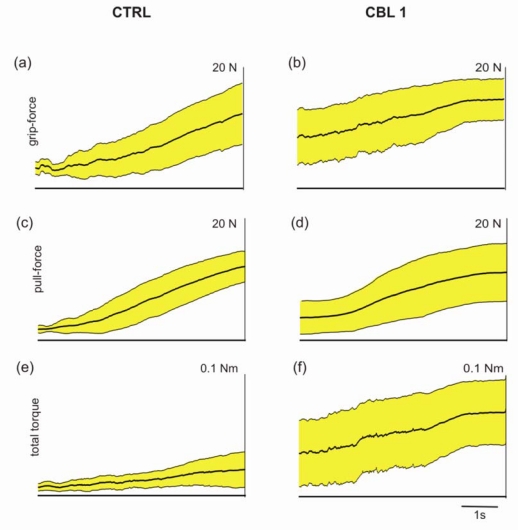
Averaged responses of grip force, pull force and torque of the healthy subject (CTRL) and the cerebellar patient (CBL1). (a, b) Averaged total grip force (thick black line) ± standard deviation (yellow area) (c, d) Averaged total pull force ± standard deviation. (e, f) Averaged total torque ± standard deviation. All recordings are aligned to the unpredictable rod release.

The economy of grip behaviour was calculated for all trials expressed by the total grip force, the pull force, and the total torque (Figure 5). The averaged data show that the patient tended to exert higher grip forces than the control subject (Figures 5a,b) but produced less pull force (Figure 5c,d) with higher variation. At URR CBL1 produced 10.3 ± 5.2 N pull force whereas CTRL produced 12.0 ± 2.3 N. This inefficiency, expressed by the production of high torques, represents losses and thus, is uneconomic (Figure 5e,f).

Taken together, our system allows insights into both the temporal coupling of grip force and pull force and into the economy of grip behaviour due to different finger position in unrestraint conditions. This latter is an essential aspect in the understanding of impaired dexterity of patients with motor disorders.

## 4. Conclusions

Standardised resistor-based pressure sensor arrays for medical and industrial applications allow the position, the size of the contact area and forces exerted by individual fingers touching an object to be determined. The system described here is approved as medical device in several countries. The size of the force-measuring film can be adapted readily to different common objects and can be attached to surfaces by adhesive tape. This makes it a suitable, easy-to-use instrument. The enlarged contact area of our system allows measurements of forces exerted by any number of fingers simultaneously without any constraints on finger position with respect to time. Hence, it allows insights into the temporal coupling between grip force and pull force as well as into the economy of grip behaviour due to different finger position under unrestraint conditions. Analysis of gripping an object in a completely natural manner reveals differences in the efficiency of grips between healthy subjects and patients with motor disorders. This is an essential aspect in basic and clinical research for detecting and understanding impaired dexterity of patients.
